# Re-Emergence of Minimal Residual Disease Detected by Flow Cytometry Predicts an Adverse Outcome in Pediatric Acute Lymphoblastic Leukemia

**DOI:** 10.3389/fonc.2020.596677

**Published:** 2021-02-05

**Authors:** Yu Wang, Yu-Juan Xue, Yue-Ping Jia, Ying-Xi Zuo, Ai-Dong Lu, Le-Ping Zhang

**Affiliations:** Department of Pediatrics, Peking University People’s Hospital, Peking University, Beijing, China

**Keywords:** acute lymphoblastic leukemia, pediatric, minimal residual disease, re-emergence, hematopoietic cell transplantation (HSCT)

## Abstract

**Purpose:**

While the role of minimal residual disease (MRD) assessment and the significance of achieving an MRD-negative status during treatment have been evaluated in previous studies, there is limited evidence on the significance of MRD re-emergence without morphological relapse in acute lymphoblastic leukemia (ALL). We sought to determine the clinical significance of MRD re-emergence in pediatric ALL patients.

**Methods:**

Between 2005 and 2017, this study recruited 1126 consecutive patients newly diagnosed with ALL. Flow cytometry was performed to monitor MRD occurrence during treatment.

**Results:**

Of 1030 patients with MRD-negative results, 150 (14.6%) showed MRD re-emergence while still on morphological complete remission (CR). Patients with white blood cell counts of ≥50 × 10^9^/L (*p* = 0.033) and MRD levels of ≥0.1% on day 33 (*p* = 0.012) tended to experience MRD re-emergence. The median re-emergent MRD level was 0.12% (range, 0.01–10.00%), and the median time to MRD re-emergence was 11 months (range, <1–52 months). Eighty-five (56.6%) patients subsequently developed relapse after a median of 4.1 months from detection of MRD re-emergence. The median re-emergent MRD level was significantly higher in the relapsed cohort than in the cohort with persistent CR (1.05% vs. 0.48%, *p* = 0.005). Of the 150 patients, 113 continued to receive chemotherapy and 37 underwent transplantation. The transplantation group demonstrated a significantly higher 2-year overall survival (88.7 ± 5.3% vs. 46.3 ± 4.8%, *p <* 0.001) and cumulative incidence of relapse (23.3 ± 7.4% vs. 64.0 ± 4.6%, *p <* 0.001) than the chemotherapy group.

**Conclusions:**

MRD re-emergence during treatment was associated with an adverse outcome in pediatric ALL patients. Transplantation could result in a significant survival advantage for these patients.

## Introduction

Acute lymphoblastic leukemia (ALL) is the most prevalent hematological malignancy in children (1). Advances in our understanding of the clinical features, immunobiological characteristics, and cytogenetic alterations associated with ALL have led to better risk stratification and risk-directed treatment of ALL patients (2, 3). In pediatric ALL, minimal residual disease (MRD) levels reflect the efficacy of chemotherapy and have shown to be the most powerful prognostic factor. While the role of MRD assessment and the significance of achieving an MRD-negative status at the end of induction and consolidation therapy have been evaluated in previous studies, there is limited evidence on the significance of MRD re-emergence without morphological relapse in ALL, in the context of sequential MRD monitoring. Our previous study showed that MRD re-emergence was an adverse prognostic factor in children at high risk of ALL (4). Pui et al. (5) and Pemmaraju et al. (6) have also reported that MRD re-emergence is associated with a poor outcome in ALL.

Flow cytometry (FCM) was explored as a less labor-intensive, less expensive, and faster MRD technique than polymerase chain reaction (PCR)-based methods and has been used extensively in pediatric ALL patients (7). Since 2005, we have monitored MRD sequentially using FCM at our institution. This study therefore aimed to determine the significance of MRD re-emergence in pediatric ALL patients after achieving an MRD-negative status.

## Materials and Methods

### Patients

Between January 2005 and December 2017, this trial recruited consecutive patients aged 0 to 18 years who were newly diagnosed with ALL. Patients with mature B-cell leukemia were excluded. The study was approved by the Ethics Committee of Peking University People’s Hospital and conducted in accordance with the Declaration of Helsinki. Written informed consent was obtained from the parents or guardians of the patients. The BOSHI Network Database (https://www.boshicloud.com), an online platform for clinical patient information management and data analysis, was used to retrieve and monitor patient data.

### Diagnosis, Minimal Residual Disease Measurement, and Risk Classification

ALL was diagnosed based on morphological, immunophenotypic, and cytogenetic evaluation using standard techniques (8, 9). Fusion transcripts of t(12;21)/*ETV6‐RUNX1*, t(1;19)/*TCF3‐PBX1*, t(9;22)/*BCR‐ABL1*, and 11q23/*KMT2A* rearrangement (*KMT2A-r*) were measured using PCR and/or fluorescence *in situ* hybridization, as previously described (10, 11).

MRD was measured using FCM, with a sensitivity of 0.01% (12, 13). The MRD monitoring schedule was planned in advance, and the scheduled time points for induction therapy were on day 15 and day 33. Patients were classified as either M1 (blast cells, <5%), M2 (5–25%), or M3 (≥25%) based on morphological evaluation. After induction therapy, MRD measurements were performed every 2–3 months during consolidation chemotherapy and every 6 months during maintenance chemotherapy (4). More frequent MRD monitoring was performed for some patients, depending on their conditions.

For initial risk stratification, we referred to the National Cancer Institute (NCI) risk group criteria (14) and cytogenetic subtypes, while the final assessment was based on treatment response and MRD levels during and after induction therapy (15) (**Supplementary Figure 1**). Standard-risk (SR) patients with an M3 marrow status on day 15 or MRD measurements of 0.01–0.99% on day 33 were upstaged to intermediate risk (IR), whereas IR patients with an M3 marrow status on day 15 were assigned high risk (HR). Patients who did not achieve complete remission (CR) upon completion of induction therapy or had MRD levels of ≥1% on day 33 or ≥0.1% on week 12 were also upstaged to HR.

### Definition

CR was defined as a percentage of leukemic blasts of <5% in the bone marrow (BM) sample reviewed at the time of peripheral blood count recovery, the absence of circulating peripheral blasts, and the absence of extramedullary disease. Relapse was defined as the presence of leukemic blasts in any extramedullary location, or in the BM at a level of ≥5%. Moreover, MRD re-emergence was defined as at least two consecutive detectable recurrences of MRD (sensitivity for positive value, ≥0.01%), despite the persistence of morphological CR. The level for MRD positivity was based on first MRD re-emergence level. After the first MRD re-emergence, a second MRD test was scheduled within the next two weeks.

### Treatment

All patients underwent a modified version of the ALL-Berlin-Frankfurt-Munster (BFM) protocol described previously (4). Briefly, the patients underwent induction therapy, including vincristine, idarubicin, cyclophosphamide, prednisone/dexamethasone, and l-asparaginase (COIPL), followed by consolidation therapy with one to two cycles of re-induction and maintenance therapy (**Supplementary Figure 1** and **Supplementary Table 1**). The consolidation chemotherapy regimen included high-dose methotrexate (HDMTX) (targeted steady-state concentration of 16 μM/L for SR patients and 24 μM/L for IR/HR patients), high-dose cytarabine (HDAra-C) (cytarabine for SR patients and cytarabine + idarubicin for IR/HR patients), and ifosfamide (IFO) (only for HR patients), which were given alternately. Re-induction comprised 1 course of COIPL for SR patients and 2 courses of COIPL for IR/HR patients. Maintenance therapy included daily mercaptopurine and weekly methotrexate. Re-induction was administered every 6 months during the consolidation chemotherapy. The scheduled consolidation chemotherapy comprised 9 rounds of HDMTX and 2 rounds of HDAra-C for SR patients, 11 rounds of HDMTX and 2 rounds of HDAra-C for IR patients, and 13 rounds of HDMTX and 3 rounds of HDAra-C for HR patients. Since 2010, for patients diagnosed with *BCR-ABL1* ALL (days 8–15 of induction), imatinib mesylate was initiated at a dose of 260 to 340 mg/m^2^/day. The total doses of idarubicin and l-asparaginase were 80 mg/m^2^ and 200 000 units/m^2^ for SR patients, and 100 mg/m^2^ and 300,000 units/m^2^ for IR/HR patients, respectively.

All patients regularly received triple intrathecal therapy to prevent central nervous system (CNS) leukemia. The total number of intrathecal therapies administered ranged from 16 in SR patients to 23 in IR/HR patients. Patients presenting with CNS leukemia received twice-weekly intrathecal chemotherapy until normalization of cerebrospinal fluid levels, after which they received weekly CNS therapy for four more doses. The total duration of treatment was 3 years for SR patients and 3.5 years for IR/HR patients. Patients in the HR group who achieved CR were offered the option of undergoing allogeneic hematopoietic cell transplantation (allo-HSCT). The transplant conditioning regimens were administered as previously described (4).

### Statistical Analysis

The outcome data used in the analysis were last updated on April 15, 2020. Overall survival (OS) was defined as the time between the date of diagnosis and the date of death due to any reason or the date of last contact. Event-free survival (EFS) was defined as the time between the date of diagnosis and the date of an event (e.g., relapse, second malignancy, death due to any reason) or the date of the last follow-up. The Kaplan-Meier method was used to estimate the survival rates, and log-rank tests were used to compare their differences. Multivariate analyses were performed using a Cox proportional hazards model. The cumulative incidence of relapse (CIR) for competing events was constructed using the Kalbfleisch-Prentice method. The OS_MRD-r_ and CIR_MRD-r_ were evaluated from the time of MRD re-emergence. Fisher’s exact test was used to compare differences between categorical variables among the groups. Logistic regression was used to evaluate factors affecting the re-emergence of MRD. R software version 4.0.1 (R Foundation for Statistical Computing, Vienna, Austria) and SPSS version 26.0 (SPSS Inc., Chicago, IL) were used for statistical analyses.

## Results

### Patient Characteristics and Treatment Outcomes

There were 1126 patients with newly diagnosed ALL during the study period in our center. Among them, 25 (2.2%) did not complete the induction treatment and lost contact, while 50 (4.4%) who were on CR without serious toxicities gave up treatment because of financial difficulties, of whom 40 were in the early intensification phase and 10 were in the consolidation phase. Ultimately, 1051 patients were enrolled in the study. At a median follow-up of 60.6 months (range, 0.8–184.5 months), the estimated 5-year OS, EFS, and CIR in the 1051 patients were 84.0 ± 1.0%, 79.0 ± 1.0%, and 17.8 ± 1.2%, respectively.

**Figure 1:**
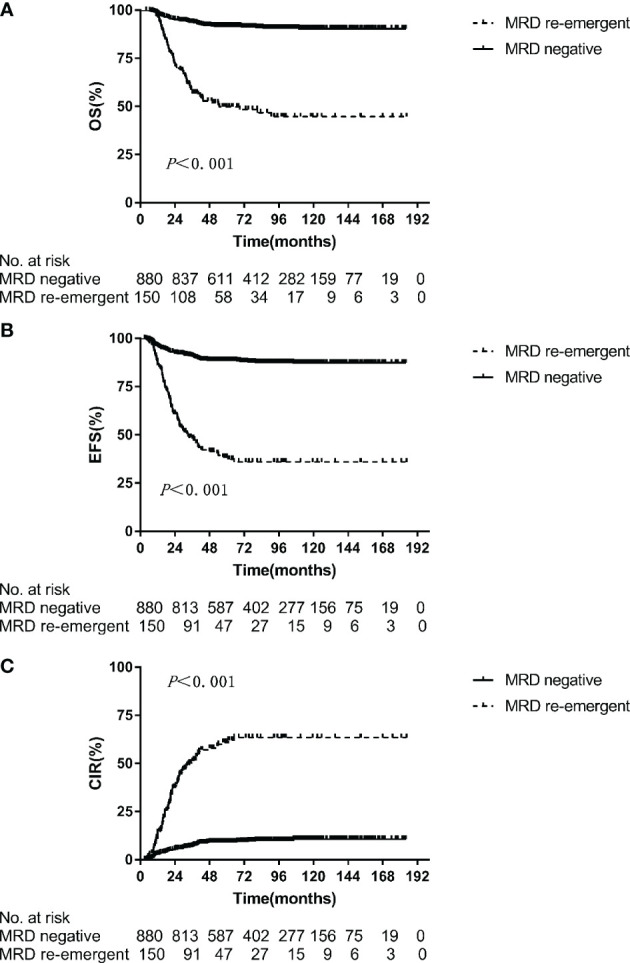
Overall survival **(A)**, event-free survival **(B)**, cumulative incidence of relapse **(C)** for MRD negative and MRD re-emergent group.

Multivariate predictors of outcome in pediatric ALL are presented in **Table 1**. In the multivariate analysis, the re-emergence of MRD during treatment was the most powerful prognostic factor for OS (*p <* 0.001, hazard ratio = 6.135), EFS (*p <* 0.001, hazard ratio = 5.848), and CIR (*p <* 0.001, hazard ratio = 7.476). The 5-year OS, EFS, and CIR for patients with re-emergent MRD were 49.8 ± 4.3%, 38.4 ± 4.2%, and 60.2 ± 4.3%, respectively. In patients with persistently MRD-negative results, the corresponding values were significantly better, at 91.7 ± 1.0%, 88.5 ± 1.1%, and 9.2 ± 1.0% (*p* < 0.001) (**Figures 1A–C**).

**Table 1:** Factors associated with outcomes in multivariate analysis in the whole group (N = 1,051).

Variable	OS	EFS	CIR
	Multivariate (*p*) HR (95% CI)	Multivariate (*p*) HR (95% CI)	Multivariate (*p*) HR (95% CI)
Age (1–10 years)	0.032 0.709 (0.518–0.971)	0.019 0.715 (0.540–0.945)	0.054 0.728 (0.527–1.005)
WBC < 50 × 10^9^/L	0.431 0.861 (0.593–1.250)	0.313 0.841 (0.624–1.177)	0.190 0.791 (0.556–1.125)
Immunophenotype(T)	<0.001 2.017 (1.365–2.982)	0.008 1.637 (1.135–2.361)	0.100 1.409 (0.935–2.123)
Day 33 MRD ≥ 0.1%	0.164 1.353 (0.884–2.070)	0.016 1.599 (1.093–2.339)	0.150 1.372 (0.894–2.106)
Week 12 MRD ≥ 0.01%	0.589 1.148 (0.696–1.893)	0.452 1.190 (0.756–1.873)	0.230 1.358 (0.8221–2.248)
Re-emergent MRD	<0.001 6.135 (4.367–8.621)	<0.001 5.848 (4.329–7.874)	<0.001 7.476 (5.405–10.309)
Risk group (high-risk)	0.001 1.795 (1.279–2.518)	0.028 1.392 (1.037–1.868)	0.120 1.289 (0.939–1.770)

MRD, minimal residual disease; WBC, white blood count; HR, hazards ratio; CI, confidence interval; EFS, event-free survival; OS, overall survival; CIR, cumulative incidence of relapse.

### Re-Emergence of Minimal Residual Disease

**Figure 2:**
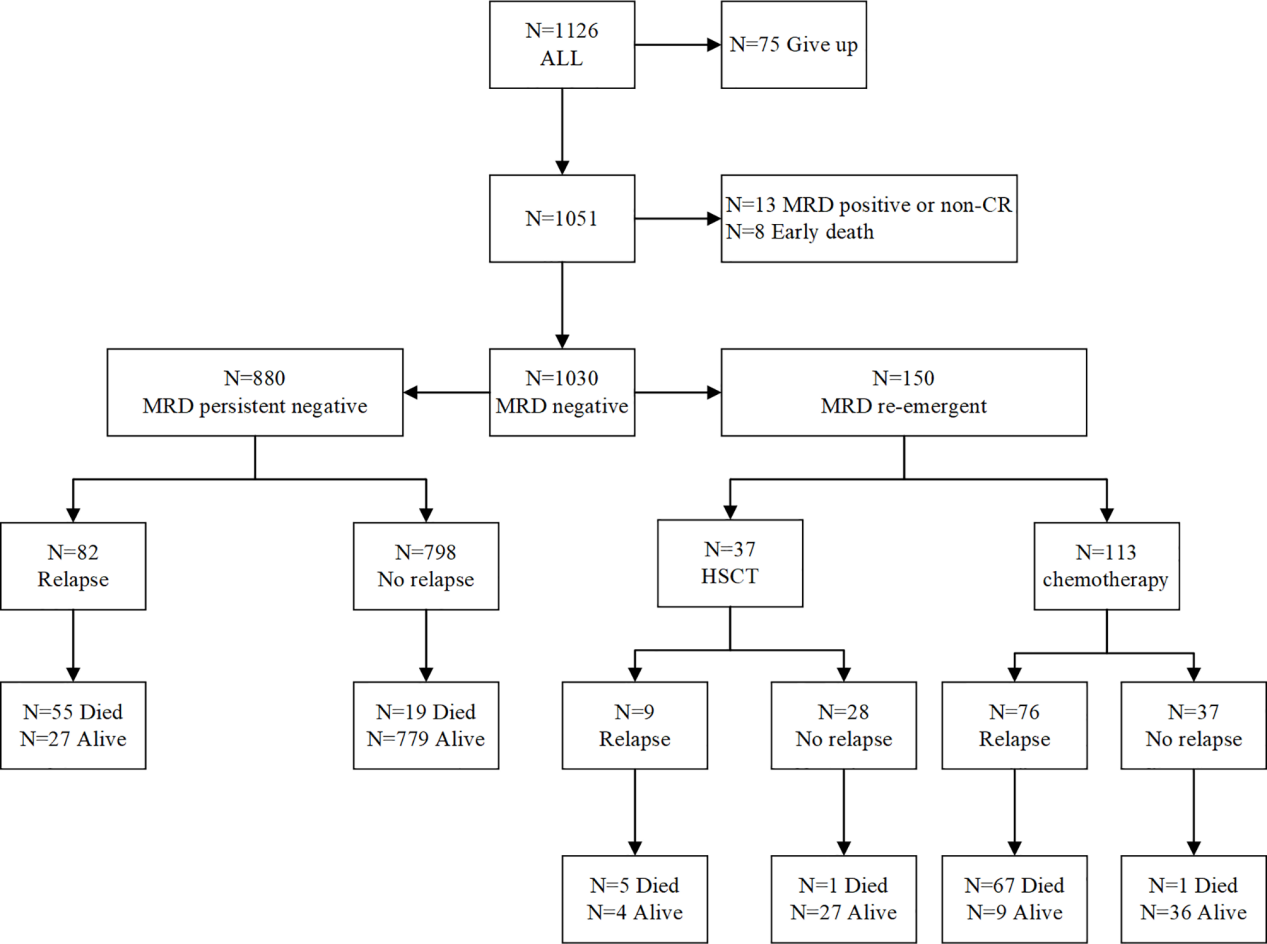
Patient disposition: study flowchart.

Among the 1051 patients, 8 died during induction therapy and 13 maintained a persistently MRD-positive status until relapse. Finally, 1030 patients achieved an MRD-negative status on BM examination. Of these patients, 150 (14.6%) ultimately developed re-emergent MRD while still on morphological CR and were the focus of this analysis. **Figure 2** depicts the study flowchart for patient disposition. Further, we analyzed the characteristics of patients with persistently MRD-negative results and re-emergent MRD (**Table 2**), and found that those with white blood cell (WBC) counts of ≥50 × 10^9^/L (hazard ratio, 1.609; 95% confidence interval [CI], 1.034–2.488; *p* = 0.033) and MRD levels of ≥0.1% on day 33 (hazard ratio, 1.908; 95% CI, 1.145–3.145; *p* = 0.012) tended to have re-emergent MRD.

**Table 2:** Characteristics of patients with MRD-negative (n=880) and MRD-re-emergent (n=150).

Variables	MRD-re-emergent		MRD-negative		*p*
	N	%	N	%	
Sex					0.997
Male	91	60.6	534	60.6	
Female	59	39.3	346	39.3	
Age(years)					0.362
<1	2	1.3	9	1.0	
1–10	93	62.0	598	67.9	
≥10	55	36.3	273	31.0	
Initial WBC (10^9^/L)					0.001
<50	102	68.0	705	80.1	
≥50	48	32.0	175	19.8	
Immunophenotype					0.029
Precursor B	122	81.3	773	87.8	
T	28	18.6	107	12.1	
Molecular subtype					0.538
*TCF3-PBX1*	9	6.0	54	6.1	
*BCR-ABL1*	9	6.0	62	7.0	
*ETV6-RUNX1*	13	8.6	143	16.2	
*KMT2A-r*	4	2.6	27	3.0	
Hyper-diploidy>50					0.830
Yes	23	15.3	129	14.6	
No	127	84.6	751	85.4	
Day 33 remission					0.014
Yes	144	96.0	871	98.9	
No	6	4.0	9	1.0	
Day 33 MRD					<0.001
<0.01%	81	54.0	651	73.9	
0.01%–0.1%	17	11.3	81	9.2	
0.1%–1%	29	19.3	73	8.2	
≥1%	21	14.0	63	7.1	
Week 12 MRD					<0.001
<0.01%	126	84.0	829	94.2	
0.01%–0.1%	10	6.6	22	2.5	
≥0.1%	14	9.3	29	3.2	
Risk group					<0.001
SR	20	13.3	301	34.2	
IR	88	58.6	417	47.3	
HR	42	28.0	162	18.4	

MRD, minimal residual disease; WBC, white blood count; SR, standard risk; IR, intermediate risk; HR, high risk.

**Figure 3:**
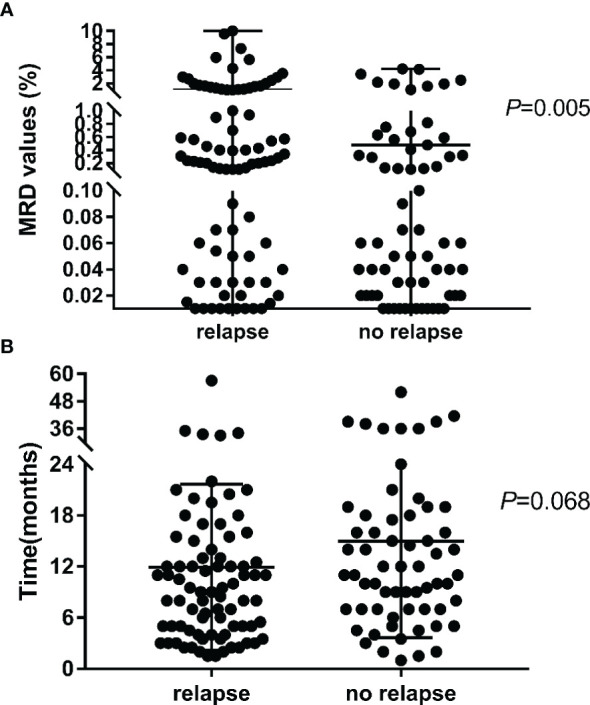
The MRD value **(A)** and duration time **(B)** for the first MRD re-emergent in the subsequent relapse and no relapse cohort.

The overall median level for MRD positivity in the 150 patients was 0.12% (range, 0.01–10.00%). The median duration from MRD negativity to MRD re-emergence was 11 months (range, <1–52 months). Eighty-five (56.6%) patients subsequently developed relapse (78 patients with BM leukemia, 4 with BM + CNS leukemia, 2 with BM + testicular leukemia, and 1 with leukemia in other extramedullary sites) after a median of 4.1 months (range, <1–47.4 months) from the detection of MRD re-emergence. Among the 150 patients with re-emergent MRD, the median level for the first MRD-positive result was significantly higher in the relapsed cohort than in the cohort with persistent CR (1.05% vs. 0.48%, *p* = 0.005) (**Figure 3A**). To further investigate the predictive role of re-emergent MRD in relapse, we performed a receiver operating characteristic (ROC) curve analysis of the first re-emergent MRD level and the actual development of relapse. It turned out that the area under the ROC curve (AUC) was 0.631 (95% CI, 0.540–0.708; *p* = 0.004). Further, we investigated the diagnostic accuracy using different MRD levels as cutoff points. The optimal cutoff point to predict relapse was 0.15%, with a sensitivity and specificity of 61.65% and 71.69%, respectively. The median duration from MRD negativity to MRD re-emergence tended to be shorter for patients who experienced a subsequent relapse, although the differences did not reach statistical significance (11.9 vs. 15.0 months, *p* = 0.068) (**Figure 3B**).

**Figure 4:**
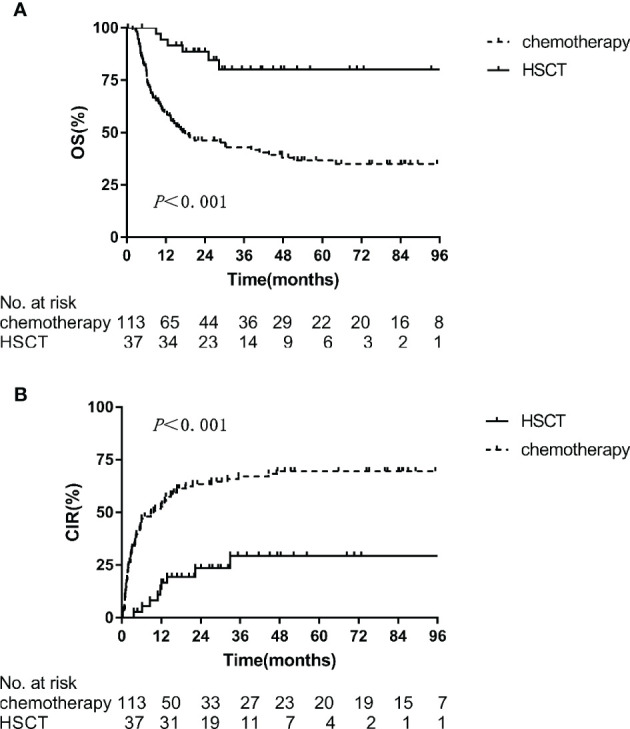
Kaplan-Meier estimates of 2-year outcomes in the HSCT and chemotherapy arms in MRD re-emergent group. **(A)** OS, **(B)** CIR.

When the OS_MRD-r_ was evaluated from the time of MRD re-emergence to the last follow-up, its median value was 20.6 months in the 150 patients. To determine if the time of MRD re-emergence had an effect on the outcome of ALL, patients were divided into two groups: <12 months and ≥12 months from MRD negativity to MRD re-emergence. No statistically significant differences in 2-year OS_MRD-r_ (60.6 ± 6.2% vs. 54.0 ± 5.5%, *p* = 0.745) and 2-year CIR_MRD-r_ (49.7 ± 6.4% vs. 56.8 ± 5.5%, *p* = 0.582) were found between the two groups. Regarding treatments prior to morphological relapse among these 150 patients, 113 patients continued to receive maintenance chemotherapy according to the specified treatment protocol and 37 underwent allo-HSCT (30 from haploidentical donors, 5 from HLA-identical sibling donors, and 2 from matched unrelated donors). Moreover, the HSCT group showed significantly better 2-year OS_MRD-r_ (88.7 ± 5.3% vs. 46.3 ± 4.8%, *p <* 0.001) and 2-year CIR_MRD-r_ (23.3 ± 7.4% vs. 64.0 ± 4.6%, *p <* 0.001) than the chemotherapy group (**Figures 4A, B**).

## Discussion

With a median follow-up of 60.6 months, this single-institution trial showed that the 5-year OS and EFS of the 1051 pediatric patients with ALL were 84.0 ± 1.0% and 79.0 ± 1.0%, which are comparable to the results of other studies (15–18). Multiple studies have established MRD detection as an independent prognostic factor for ALL and have demonstrated that achievement of an MRD-negative status could lead to better clinical outcomes (3, 19, 20). In our study, we applied FCM for sequential post-remission MRD measurement and found that patients with higher end-induction MRD levels (≥0.1%) and positive levels of MRD on week 12 (≥0.01%) exhibited a worse EFS and OS. However, in the multivariate analysis of the whole cohort, MRD at any particular time point did not show a strong prognostic significance. We calculated that there may be two reasons for this result. First, the risk stratification of the patients in this study was adjusted based on the MRD level at end-induction and week 12. Therefore, risk stratification-oriented treatment may affect the results. Second, MRD may not show a strong prognostic significance on the context of MRD-guided therapy and MRD alone was not sufficient to fully predict outcomes. The significance of MRD on treatment outcomes varied depending on leukemia subtypes and measurement time, such as different genotypes. Meanwhile, re-emergent MRD during treatment was the most powerful adverse prognostic indicator, even after adjusting for other risk factors. The 5-year OS (91.7 ± 1.0% vs. 49.8 ± 4.3%, *p <* 0.001) and EFS (88.5 ± 1.1% vs. 38.4 ± 4.2%, *p* < 0.001) were significantly better in the persistently MRD-negative group. This obvious survival gap strongly confirmed the poor prognostic significance of MRD re-emergence in ALL.

In this study, 14.6% (150/1030) of pediatric ALL patients experienced MRD re-emergence while still on morphological CR. Patients with a high leukemia burden (WBC ≥ 50 × 10^9^/L) and a poor response to early treatment (MRD levels ≥ 0.1% on day 33) were prone to MRD re-emergence, which indicates the need for further strengthening MRD monitoring in these patients. Several previous investigations demonstrated the clinical potential and prognostic value of FCM- or PCR-based MRD quantification in the post-remission setting, producing lead times from clinical relapse of 3.6 to 4.1 months (6, 21). In our analysis, 85 (56.6%) patients subsequently developed relapse after a median of 4.1 months from the detection of re-emergent MRD, and this finding was consistent with those of previous studies. Additionally, a strong correlation was observed between re-emergent MRD levels and clinical relapse, suggesting that a higher re-emergent MRD level (cutoff, 0.15%) may signify an impending relapse. It was worth mentioning that a total of five patients had a re-emergence of MRD > 2%, but never developed a morphologic relapse. One of the patients had a large deletion of IKZF gene. He started taking tyrosine kinase inhibitors after MRD recurrence, and continued to survive disease-free. As of the last follow-up date, he had been followed up for 61.6 months. The other four patients all chose further transplantation rescue treatment after MRD recurrence, and all of them survived disease-free.

As re-emergent MRD can reliably predict clinical relapse, we should monitor MRD sequentially to expand the time window for a more effective preemptive treatment against a potential relapse (22, 23). In this retrospective study, patients with re-emergent MRD were given the choice between HSCT or chemotherapy according to their preference. The results showed that the HSCT group had a significantly higher survival advantage than the chemotherapy group. Re-emergent MRD may be a group of residual leukemia cells that are out of the detection range of FCM and resistant to chemotherapy (3, 20). Although the intensification of chemotherapy may not fully eliminate re-emergent MRD, the strong graft-versus-leukemia effect of HSCT may help (24). However, the outcome of the HSCT group in this study was unsatisfactory, highlighting the urgent need for novel, less toxic strategies and enrollment of subjects in clinical trials specifically designed for ALL patients with MRD persistence or re-emergence.

This study has a few limitations. It is a retrospective single-center study without predetermined enrollment criteria. Limited by the availability of donors and patients’ preference of whether to undergo HSCT, we were unable to define the indications of transplantation in advance. Furthermore, technical constraints such as low tumor burden, immunophenotypic shifts, and clonal selection may have contributed to a decreased sensitivity of measurements, leading to more false-negative results, as suggested by the occurrence of relapse in patients with negative FCM-MRD findings (25). Recent studies have described a highly sensitive next-generation sequencing platform to monitor MRD and have observed the conversion of an MRD-negative status to a positive one as early as 25.6 weeks prior to clinical relapse (21). Besides, patients in this study did not undergo a unified treatment escalation after the recurrence of MRD, because although multiple studies have confirmed the poor prognosis of MRD re-emergence, the current international standards have not yet reached a consensus on the treatment of risk escalation after the recurrence of MRD. However, we believe that the findings of this study will provide more powerful evidence to support the future treatment options for patients with re-emergent MRD.

In conclusion, this study revealed that MRD re-emergence at any time after induction and consolidation therapy was associated with relapse in pediatric ALL patients. We also found that patients with re-emergent MRD could benefit from HSCT, reflecting the necessity of sequential MRD monitoring for better risk stratification and earlier preemptive therapies against impending relapse, thus potentially improving outcome for pediatric B-ALL. Prospective studies on sequential MRD monitoring coupled with less toxic strategies such as chimeric antigen receptor T cell therapy designed to eradicate MRD are warranted to address the unmet medical needs of pediatric ALL patients.

## Data Availability Statement

The original contributions presented in the study are included in the article/**Supplementary Material**, further inquiries can be directed to the corresponding authors.

## Ethics Statement

Written informed consent was obtained from the individual(s), and minor(s)’ legal guardian/next of kin, for the publication of any potentially identifiable images or data included in this article.

## Author Contributions

Material preparation and data collection were performed by Y-PJ, Y-XZ, and A-DL. Data analysis was performed and the first draft of the manuscript was written by YW and Y-JX, and they contributed equally to this work. L-PZ designed the research and was the chief person in charge of the manuscript. All authors commented on previous versions of the manuscript. All authors contributed to the article and approved the submitted version.

## Funding

This work was supported by the Foundation of 2018 Beijing Key Clinical Specialty Construction Project-Pediatrics (2199000726).

## Conflict of Interest

The authors declare that the research was conducted in the absence of any commercial or financial relationships that could be construed as a potential conflict of interest.
