# Supplementary material for: Re-Emergence of Minimal Residual Disease Detected by Flow Cytometry Predicts an Adverse Outcome in Pediatric Acute Lymphoblastic Leukemia
Source: Front Oncol. 2021 Feb 5;10:596677. doi: 10.3389/fonc.2020.596677 (PMC7892594; doi:10.3389/fonc.2020.596677)
Supplement: Supplementary file 1 [file DataSheet_1.docx]

***Supp.* Figure 1 Flow chart of risk stratification and treatment protocol.**

**Supplemental Table 1 Modified ALL-BFM protocol in our institution**

| **Treatment element/drug** | **Single or daily dose** | **Days** |
| --- | --- | --- |
| **Induction and re-induction** |  |  |
| **COIPL** |  |  |
| VCR | 1.5mg/m^2^ (max:2mg) | 1,8,15,22 |
| CTX | 1g/m^2^ | 1 |
| DEX or Pred | 10mg/m^2^(max:10mg) or 60mg/m^2^(max:60mg) | 1-28 |
| IDR | 8-10mg/m^2^ | SR:8;  IR/HR: 1,8 |
| L-asp | 10000U/m^2^ | 15,17,19,21,23,25,27,29,31,33 |
| **Consolidation** |  |  |
| **HDMTX×2**  HDMTX  VCR  IDR  **HDMTX**  HDMTX  VCR  **HDAra-C**  HDAra-C  IDR (IR, HR only)  **IFO (HR only)**  IFO  VCR | 2.5-3.5g/m^2^  1.5mg/m^2^ (max:2mg)  8-10mg/m^2^  2.5-3.5g/m^2^  1.5mg/m^2^ (max:2mg)  2g/m^2^  8-10mg/m^2^  1g/m^2^  1.5mg/m^2^ (max:2mg) | 1,22  1,8,15,22  SR:8;  IR/HR: 8,10  1  1  1-3  2,3  1-5  1 |
| VP-16  **Maintenance therapy**  6-MP(oral)  MTX(IM) | 100mg/m^2^  50mg/m^2^  20mg/m^2^ | 3-5  Once a day  Once a week |

SR, standard risk; IR, intermediate risk; HR, high risk; DEX, dexamethasone; Pred, prednisone; VCR, vincristine; CTX, cyclophosphamide; IDR, idarubicin; L-asp, native Escherichia coli L-asparaginase; HDMTX, high-dose methotrexate; HDAra-C, high-dose cytarabine; IFO, ifosfamide; VP-16, etoposide; 6-MP, mercaptopurine; IM, Intramuscular injection.
